# Association between Inflammatory Markers and Psychometric Scores in Patients with Hidradenitis Suppurativa

**DOI:** 10.3390/jcm13195795

**Published:** 2024-09-28

**Authors:** Aikaterini I. Liakou, Nikolaos Rotsiamis, Andreas G. Tsantes, Eleni Routsi, Natalia Rompoti, Petros Ioannou, Alexandra Mpakosi, Lydia Tsamtsouri, Efthymia Agiasofitou, Ourania Kotsafti, Stefanos Bonovas, Alexander Katoulis, Evangelia Papadavid, Dimitris Rigopoulos

**Affiliations:** 1 1st Department of Dermatology-Venereology, “Andreas Sygros” Hospital, Medical School, National and Kapodistrian University of Athens, 16121 Athens, Greece; routsie@gmail.com (E.R.); natalia.rompoti@gmail.com (N.R.); efi.agiasofitou@yahoo.com (E.A.); raniakotsafti@gmail.com (O.K.); 2 2nd Department of Dermatology-Venereology, “Attikon” University Hospital, Medical School, National and Kapodistrian University of Athens, 12462 Athens, Greece; nickrotsiamis@gmail.com (N.R.); alexanderkatoulis@yahoo.co.uk (A.K.); papadavev@yahoo.gr (E.P.); dimitrisrigopoulos54@gmail.com (D.R.); 3 Laboratory of Haematology and Blood Bank Unit, “Attikon” Hospital, Medical School, National and Kapodistrian University of Athens, 12462 Athens, Greece; andreas.tsantes@yahoo.com (A.G.T.); tsamtsouri96@gmail.com (L.T.); 4 Microbiology Department, “Saint Savvas” Oncology Hospital, 11522 Athens, Greece; 5 School of Medicine, University of Crete, 71003 Heraklion, Greece; 6 Department of Microbiology, General Hospital of Nikaia “Agios Panteleimon”, 18454 Piraeus, Greece; alexiabakossi@yahoo.gr; 7 Department of Biomedical Sciences, Humanitas University, Pieve Emanuele, 20072 Milan, Italy; sbonovas@gmail.com; 8 IRCCS Humanitas Research Hospital, Rozzano, 20089 Milan, Italy

**Keywords:** Hidradenitis, CRP, ESR, UCLA, DLQI, HADS, inflammatory markers, psychometric scores

## Abstract

**Background**: Hidradenitis suppurativa (HS) is a chronic inflammatory skin disorder with a significant impact on the quality of life of affected patients. This study aimed to correlate serum inflammatory markers with specific tools assessing quality of life, emotional well-being, and loneliness, such as the Dermatology Life Quality Index (DLQI), Hospital Anxiety and Depression Scale (HADS), and the UCLA Loneliness Scale. **Methods**: A pilot observational study including 37 patients with HS was conducted. Inflammatory serum markers, including C-reactive protein (CRP) and Erythrocyte Sedimentation Rate (ESR), were evaluated at baseline, 3 months, and 6 months later. Psychometric scores were also evaluated at the same study intervals. **Results**: DLQI was correlated with ESR at baseline (Spearman’s rho = 0.35, *p* = 0.03), indicating that poorer quality of life is associated with changes in this serum marker. Disease activity, as reflected by inflammatory markers, was associated with significant psychological burden. Specifically, a worse DLQI score was associated with higher ESR (estimate β = 0.14, 95% confidence interval [CI]: 0.05–0.22; *p* = 0.001) and higher CRP level (estimate β = 0.25, 95% CI: 0.02–0.48; *p* = 0.02). Similarly, a worse UCLA score was associated with higher ESR (estimate β = 0.11, 95% CI: 0.02–0.20, *p* = 0.01). **Conclusions**: Our study results underline the close relation between systemic inflammatory markers and clinical severity together with psychological burden in HS patients, as indicated by the significant association that was revealed between ESR/CRP and poorer psychometric scores. However, further research is warranted to validate these findings.

## 1. Introduction

Hidradenitis suppurativa (HS) is a chronic inflammatory skin condition primarily affecting the intertriginous areas such as the axillae, groin, buttocks, and inframammary folds. It is characterized by recurrent painful nodules, abscesses, sinus tracts, and scars, which can significantly impair the quality of life of the affected patients due to its chronicity, debilitating symptoms, and limited treatment options [1,2]. The etiology of HS is multifactorial: chronic inflammation, dysregulation of the immune system, abnormal hair follicle biology, genetic susceptibility, and dysbiosis of the skin microbiome are some of the contributing factors to the development and persistence of HS symptoms [3,4]. The disease manifests typically with flares and remissions throughout a patient’s life, while it has been recently subclassified into two phenotypes: the inflammatory (active) phenotype and the non-inflammatory (non-active) phenotype [5]. Cases of exacerbation and new onset of HS have been recently reported following COVID-19 vaccination, further supporting the immune-mediated inflammatory nature of the disease [6].

Laboratory evaluation of the disease’s activity is mainly based on commonly used serum markers for evaluation of systemic inflammation, such as C-reactive protein (CRP) and erythrocyte sedimentation rate (ESR). CRP is an acute-phase protein produced in response to inflammation, while ESR measures the rate at which red blood cells settle in a tube over a specified time- period and is a non-specific marker of inflammation that can be influenced by various factors. These markers are particularly relevant in the context of HS, given the chronic inflammatory state associated with the condition [7,8,9]. CRP exhibits a short half-life and is responsive to cytokine-mediated inflammation, distinguishing it from ESR, which reflects a broader range of physiological changes, including anemia and leukocytosis. This diversity in ESR’s response aligns with its potential superiority in gauging HS severity, as evidenced by its sensitivity in detecting milder inflammatory states compared to CRP [10]. Other potential new markers of severity in HS have also been studied. Iannone et al. recommended an assessment of serum amyloid A (SAA) levels to monitor therapeutic response in patients with HS in order to prevent the disease’s flare and potential complications. Elevated levels of inflammation, marked by increased SAA, are linked to a higher risk of complications, including soft tissue infections, sepsis, extensive scarring leading to functional limitations, anemia, hypoproteinemia, nephrotic syndrome, joint disorders, and systemic amyloidosis [11].

The disease has a significant impact on the quality of life in patients with HS. Work productivity and leisure activity are often found to be impaired in patients with HS, leading to a significant psychosocial burden. Affected patients can suffer from depression and anxiety and often feel socially isolated because of their disease. According to Senthilnathan et al., the level of negative feelings is high in HS patients compared to healthy adults. [12]. The University of California Los Angeles (UCLA) Loneliness Scale, the Hospital Anxiety and Depression Scale (HADS), and the Dermatology Life Quality Index (DLQI) are commonly used psychometric instruments to evaluate psychological well-being and quality of life in patients with HS. The UCLA Loneliness Scale is a commonly used measure of loneliness, originally released in 1978 as a 20-item scale [13,14]. The Hospital Anxiety and Depression Scale (HADS) is a self-reported questionnaire that is widely used to assess symptoms of anxiety and depression in medical and non-psychiatric populations. The HADS consists of two subscales: one for anxiety symptoms and the other for depression symptoms [15,16]. Last, the Dermatology Life Quality Index (DLQI) is a widely utilized dermatology-specific questionnaire that evaluates the impact of skin diseases on a patient’s quality of life. It consists of ten items that cover different aspects, including symptoms and feelings, daily activities, leisure, work and school, personal relationships, and treatment [17,18].

The study aimed to evaluate the psychological impact of the disease in patients with HS through assessment of certain psychometric scores, including UCLA, HADS, and DLQI, and correlate serum inflammatory markers such as CRP and ESR with these psychometric scores.

## 2. Materials and Methods

A pilot observational longitudinal study was performed from November 2021 to November 2023 at the HS outpatient clinic of the Second Department of Dermatology-Venereology, in “Attikon” University Hospital, Athens. Written consent was obtained from all patients, while the study was approved by the Institutional Review Board (Reference No. 1891 and 3 November 2016) of the “Attikon” University Hospital. Eligible patients were considered those who were ≥18 years old, with a history of HS for more than 1 year. Patients with flu-like symptoms or systemic infection within 15 days before evaluation and those who had undergone surgery within 4 weeks were excluded to prevent potential confounding effects, as these conditions may temporarily elevate inflammatory markers independent of HS activity. Due to the limited size of our sample, we did not have the opportunity to include patients diagnosed with psychiatric disorders. As a result, the study cohort consisted exclusively of individuals without any known psychiatric comorbidities. In all patients, inflammatory serum markers, including CRP and ESR, were measured at three time points: at the beginning of the study, at 3 months, and at 6 months. During the 6-month follow-up period, patients in our study received a combination of local and systemic treatments, which included both antibiotic and biological therapies. Specifically, patients with Hurley stage II were on antibiotic treatment, while patients with Hurley stage III were receiving biological treatment.

At the same time points, all patients answered the following three questionnaires: UCLA, DLQI, and HADS. Clinical evaluation of patients was performed based on the Hurley score and the International Hidradenitis Suppurativa 4 (IHS4) score. Based on Hurley score, patients are classified into three stages, while based on IHS4 score, disease severity is characterized as mild, moderate, or severe. Data regarding demographics (such as age, gender, smoking, and body mass index [BMI]) and HS scores (Hurley score, IHS4) were collected, while data regarding the type of treatment (adalimumab, doxycycline or clindamycin plus rifampicin) were also registered. Demographics and laboratory findings were compared based on the Hurley stage, and the association between psychometric tests and inflammatory markers was estimated.

### Statistical Analysis

Statistical analysis included descriptive statistics of the study population. Data are presented as median and interquartile range (IQR) for continuous variables and as frequency and percentage for categorical variables. Comparisons of continuous and categorical variables between patients on different Hurley stages were performed using the Wilcoxon rank-sum test and the chi-square test, respectively. The Spearman’s correlation test was used to examine the association between inflammatory markers and psychometric scores at the three time points separately. Linear mixed effects models (LMMs) were employed to assess the association between psychometric scores and inflammatory markers, with random intercepts included for each subject to account for multiple observations per subject. Univariable analyses were conducted with UCLA, DLQI, and HADS as the dependent variables and CRP and ESR as the independent variables, respectively. Additionally, multivariable LMMs were used to evaluate the impact of disease activity, as reflected by inflammatory markers, on psychological state, adjusting for gender, treatment, BMI, IHS4, Hurley stage, and smoking status. Statistical analysis was conducted using the R programming language (version 4.0.3), and a *p*-value of less than 0.05 was considered statistically significant.

## 3. Results

### 3.1. Baseline Characteristics

Overall, 43 patients were screened as eligible for the study. Five patients were excluded due to systemic infection within 15 days prior to the study onset, while one patient was also excluded because he underwent surgery 3 weeks before the evaluation. Therefore, 37 participants were finally included in the study: 18 (49%) patients were on stage II, and 19 (51%) patients were on stage III based on the Hurley score. All 37 patients completed the 6-month follow-up, resulting in a 100% response rate. The median age of the included patients was 37 years (IQR: 28–49), and the median BMI was 30 kg/m^2^ (IQR: 28–35), while most participants were women (*n* = 23; 62%) and active smokers (*n* = 25; 68%). Among the included patients, 20 (54%) patients were treated with adalimumab, while the remaining 17 (46%) patients were receiving antibiotics such as doxycycline or clindamycin plus rifampicin. Most patients who were receiving adalimumab were on Hurley stage III (*n =* 18; 90%), while most patients receiving antibiotics were on Hurley stage II (*n =* 16; 94%).

Moreover, based on the IHS4 score, the severity of the disease was characterized as mild in 6 (16%) patients, as moderate in 18 (49%) patients, and as severe in 13 (35%) patients. Our focus is on understanding the psychological impact of HS in relation to inflammation and disease severity. The IHS4 score, while correlated with the Hurley system, is essential in quantifying inflammation more precisely, which is critical for assessing its psychological effects.

The type of treatment (biologic therapy vs. antibiotics) differed between patients on Hurley stage II and stage III (*p* < 0.001), while smoking was more common in patients on stage III compared to those on stage II (43% vs. 24%, *p* = 0.03). Patients on Hurley stage III were older than patients on stage II (medians: 47 vs. 29 years, *p* < 0.001), while they also had a higher BMI (medians: 33 vs. 29 kg/m^2^, *p* = 0.04). Finally, IHS4 score was higher in patients on stage III compared to patients on stage II (*p* < 0.001; Table 1).

### 3.2. Inflammatory Markers and Psychometric Scores

Regarding the inflammatory markers, the baseline evaluation revealed that patients on Hurley stage III had higher ESR than those on stage II (medians: 29 vs. 13 mm/h, *p* = 0.004), while CRP levels were similar (medians: 8 vs. 5 mg/dl, *p* = 0.19; Table 1). The median ESR was 18 mm/h (IQR: 8–26) at 3 months and 15 mm/h (IQR: 7–21) at 6 months, with ESR values being higher for patients on stage III compared to those on stage II at both 3 months (medians: 26 vs. 12 mm/h, *p* = 0.001) and 6 months (medians: 19 vs. 10 mm/h, *p* = 0.005), respectively (Table 2). The median CRP value was 4 mg/L (IQR: 2–6) at 3 months and 3 mg/L (IQR: 2–6) at 6 months, and it was similar for the two Hurley stages at both time intervals (*p* = 0.41 and *p* = 0.14). Regarding the psychometric scores, the baseline DLQI, HADS, and UCLA scores were similar between the two Hurley stages (*p* = 0.49, *p* = 0.69, and *p* = 0.68, respectively; Table 1). The median DLQI was 5 (IQR: 2–15) at 3 months, and 5 (IQR: 2–10) at 6 months, while no significant difference was found between patients with different Hurley stages at both intervals (*p* = 0.23 and *p* = 0.29, respectively; Table 2).

Similarly, patients on Hurley stage II and III did not differ in terms of the HADS (*p* = 0.32 and *p* = 0.61 for 3 and 6 months, respectively) and the UCLA score (*p* = 0.59 and *p* = 0.29 for 3 and 6 months, respectively).

### 3.3. Association of Psychometric Scores and Inflammatory Markers

A positive correlation between DLQI and ESR values was found at baseline (Spearman’s rho = 0.35, *p* = 0.03; Table 3), indicating that patients’ quality of life based on this index is associated with changes in this serum marker which reflects systemic inflammation; thus disease activity. The association between disease activity, as reflected by the inflammatory markers, and a patient’s quality of life and psychological state based on DLQI and UCLA scores is further supported by the univariable and multivariable analyses (Table 4).

Specifically, the multivariable analysis revealed that worse quality of life based on DLQI score was associated with higher ESR (estimate β = 0.14, 95% confidence interval [CI]: 0.05–0.22; *p* = 0.001) and higher CRP level (estimate β = 0.25, 95% CI: 0.02–0.48; *p* = 0.02). Similarly, a worse UCLA score was associated with higher ESR (estimate β = 0.11, 95% CI: 0.02–0.20, *p* = 0.01), indicating that increased disease activity has a significant psychological burden. The scores obtained in our study for the DLQI, HADS, and UCLA scales are consistent with those reported in the literature for similar dermatological conditions. This supports the reliability of these scales in assessing the psychological impact of HS.

## 4. Discussion

To the best of our knowledge, this is the first study associating inflammatory markers with clinical severity and psychometric scores in patients with HS. Our results are based on the existing literature underscoring the impact of systemic inflammation on disease severity and quality of life in patients with HS [19]. Notably, our study extended our knowledge of HS by explicitly linking inflammatory markers with psychometric outcomes, highlighting the multifaceted nature of HS. Specifically, the association of ESR with the UCLA score indicated that higher inflammation levels may contribute to increased feelings of loneliness among HS patients. Similarly, DLQI scores were positively associated with both ESR and CRP levels, implying that inflammation exacerbates the physical and emotional burden of HS. Therefore, systemic inflammation—besides correlating with disease severity—is a major determinant that drastically influences the quality of life and mental health of patients. The reliability of the DLQI, HADS, and UCLA questionnaires in HS has not been extensively studied. However, a recent scoping review highlights that available data primarily address the DLQI in HS populations. There is a substantial body of evidence from other dermatological conditions, where these tools have been shown to be highly reliable and valuable in assessing the psychological and quality-of-life impacts of disease. Given the overlap in psychosocial burden across dermatologic diseases, it is reasonable to expect that these instruments would be equally valid in the HS context. Future research should aim to address this gap by directly validating these questionnaires in HS populations to strengthen the evidence base [20].

The potential link between inflammation and increased feelings of loneliness, as well as the impact on quality of life, raises an important question about whether there is a common pathway connecting HS, depression, and autoimmune responses. This hypothesis merits further investigation through larger studies to explore if treating inflammation could concurrently address depressive symptoms or if targeted treatment for depression could improve HS outcomes. Understanding these pathways could pave the way for more integrated and effective treatment strategies in the future.

Previous research has linked serum inflammation markers with HS activity, indicating that several biomarkers, such as serum IL-6, CRP, and ESR, can be used as reliable markers for assessing disease severity [21,22,23]. Jiménez-Gallo et al. compared several laboratory parameters between patients with HS and healthy controls and found that patients with HS had elevated levels of serum proinflammatory cytokines, CRP, and ESR [21]. Moreover, the levels of these cytokines were associated with the clinical severity, indicating a higher systemic inflammatory burden in more severe cases of HS. In another study, Hessam et al. showed that the results of routine laboratory studies such as CRP, white blood cell count, and neutrophil count were also associated with the disease severity [22]. The authors found that CRP levels and neutrophil count were correlated to a modified Hidradenitis Suppurativa score, suggesting that these laboratory markers are significant independent predictors for a more severe Hurley stage. The authors concluded that certain biomarkers such as CRP, which are widely available and have a low cost, should be incorporated into the currently available clinical scoring systems for HS. In another recent study, Solak et al. compared more specific laboratory parameters, including the neutrophil/lymphocyte ratio (NLR), the systemic immune-inflammation index (SIII), and the pan-immune-inflammation value (PIV) between HS patients and healthy individuals, to determine their correlation with disease severity [23]. All these parameters differed between HS patients and healthy controls, while they were also positively associated with the disease severity. Based on their findings, the authors of this study suggested that NLR, SIII, and PIV values can be utilized as simple and cost-effective tests to monitor disease activity and severity in HS patients. Last, Gambichler et al. also evaluated a large panel of laboratory indicators of systemic inflammation, such as NLR, platelet/lymphocyte ratio (PLR), and platelet/neutrophil ratio (PLR), for their association with disease activity in patients with HS [24]. The authors reported that although PIV and SII were significantly higher in HS patients, the PLR and monocyte/lymphocyte ratio (MLR) were significantly lower in HS patients. Notably, the PIV was the only parameter that was significantly associated with HS severity. In line with the results of all these studies, we also found that patients on Hurley stage III had higher ESR than those on stage II. However, CRP values were similar in both stages, which could potentially indicate that ESR is a more suitable biomarker than CRP for monitoring disease severity. Although there is a large body of evidence linking disease severity to several biomarkers, there is a need for larger and more comprehensive diagnostic studies to evaluate the diagnostic accuracy of these parameters and to set the optimal cutoff values with the highest sensitivity and specificity to identify patients at higher risk for a more severe disease [24].

There is a large body of evidence associating clinical severity with the psychosocial impact in patients with HS. Jfri et al. evaluated certain severity scores, such as the IHS4 and the Severity Assessment of Hidradenitis Suppurativa (SAHS) score in patients with HS [25]. The authors found that patients with IHS4 score ≥ 3.5 were 9.4 times more likely to experience moderate to severe impairment of quality of life based on the DLQI. Similarly, patients with SAHS ≥ 5.5 were 6.2 times more likely to experience moderate to severe impairment of quality of life. The IHS-4 and SAHS scores demonstrated good diagnostic capacity for predicting moderate to severe impact on quality of life (Area under the curve: 0.787 for IHS-4 and 0.733 for SAHS). Moreover, a SAHS cutoff score of 5.5 showed a sensitivity of 72% and a specificity of 71% for the prediction of moderate to severe impairment of quality of life. In a recent systematic review including 58 observational studies that evaluated the overall burden of HS, DLQI was found to be the most widely used tool for assessing the psychological burden of HS [26]. The reported mean DLQI score was between 8.4 and 16.9, indicating a significant impact on the quality of life of these patients, with higher DLQI scores being reported in patients with a more severe disease, and among female patients. A negative impact of HS on psychological well-being based on patient-reported outcome (PRO) scores related to depression and anxiety was also revealed. Notably, a high rate of patients reported sexual dysfunction, with the larger impact being recorded in women than men. Work productivity and leisure activity were also consistently found to be impaired in patients with HS [26]. In our study, the median DLQI score was 16 at baseline and dropped to 5–9 in the following study period, also highlighting the significant impairment of quality of life in patients with HS. In another study, Montero-Vilchez et al. evaluated the quality of life in patients with HS also using DLQI. The authors of this study reported that the severity of symptoms correlated with poorer quality of life, while also certain aspects of quality of life, such as sexual distress, anxiety, depression, and sleep, were negatively associated with symptom severity. Finally, Montero-Vilchez et al. conducted a comprehensive meta-analysis evaluating the impact of HS signs and symptoms on quality of life, synthesizing data from 17 studies including 4929 patients [27]. The findings of this meta-analysis also underscored the significant correlation between the intensity of HS symptoms and poorer overall quality of life. The authors highlighted that despite the chronic and debilitating nature of HS symptoms characterized by painful lesions, pruritus, malodor, and suppuration, the assessment of their severity in clinical practice remains sporadic [27].

There are certain limitations of this study that need to be addressed. First, although this is a pilot study evaluating the association between serum markers and psychological impact in patients with HS, without any similar studies in the literature, the small number of patients is a certain limitation. Therefore, further studies with larger populations are needed to validate our results. Second, due to the small number of participants, our study population does not reflect the whole spectrum of disease severity since only patients on Hurley stage II and III were included, while there were not any patients on stage I. This could be attributed to the fact that patients with very mild symptoms that would be classified as stage I do not seek medical treatment. Last, the laboratory evaluation of systemic inflammation included a rather limited panel, including only CRP and ESR, while more comprehensive studies were not performed. Although we aimed to evaluate only tests that are widely available at a low cost, future studies should be conducted by evaluating a wider set of laboratory studies.

In conclusion, HS is a complex inflammatory disorder with a significant impact on the physical and psychological well-being of affected patients. Psychosocial factors can affect the natural history of the disease and require a holistic treatment approach targeting both the inflammatory and psychosocial aspects of patients with HS. Our study results underline the close relationship between systemic inflammation, as reflected by two inflammatory markers, and psychosocial status in HS patients, as indicated by the significant association revealed between serum ESR/CRP and poorer psychometric scores. Future research is needed to further investigate this association and assess potential interventions that could aid in reducing inflammation, resulting in improved mental health outcomes in HS patients. Understanding the relationship between systemic inflammation and mental well-being in HS patients could inform a more comprehensive treatment approach. Regular monitoring of psychosocial factors while managing systemic inflammation may help improve both physical and mental health outcomes, emphasizing the need for interdisciplinary care in HS management.

## Figures and Tables

**Table 1:** Baseline characteristics of the 37 patients with HS by Hurley stage.

	Hurley II (*n =* 18)	Hurley III (*n =* 19)	Total (*n =* 37)	*p*-Value
Gender, N (%)				0.18
Female	9 (24)	14 (38)	23 (62)	
Male	9 (24)	5 (13)	14 (38)	
Treatment, N (%)				**<0.001**
Adalimumab	2 (5)	18 (49)	20 (54)	
Other	16 (43)	1 (3)	17 (46)	
Smoking, N (%)				**0.03**
Yes	9 (24)	16 (43)	25 (68)	
No	9 (24)	3 (8)	12 (32)	
IHS4, N (%)				**<0.001**
Mild	6 (16)	0	6 (16)	
Moderate	12 (32)	6 (16)	18 (49)	
Severe	0	13 (35)	13 (35)	
Age (yrs.), median (IQR)	29 (24–21.3)	47 (34–58)	37 (28–49)	**0.001**
BMI (kg/m^2^), median (IQR)	29 (27–30)	33 (29–36)	30 (28–35)	**0.04**
ESR (mm/h), median (IQR)	13 (8–24)	29 (15–38)	23 (10–29)	**0.004**
CRP (mg/L), median (IQR)	5 (3–8)	8 (3–13)	7 (2–9)	0.19
DLQI, median (IQR)	10 (4–23)	15 (5–23)	13 (4–22)	0.49
HADS, median (IQR)	8 (5–11)	9 (5–11)	9 (5–9)	0.69
UCLA, median (IQR)	34 (30–39)	39 (31–47)	36 (30–45)	0.68

Abbreviations: IHS4, International Hidradenitis Suppurative Severity Score System; BMI, body mass index; ESR, erythrocyte sedimentation rate; CRP, C-reactive protein; DLQI, Dermatology Life Quality Index; HADS, Hospital Anxiety and Depression Scale; UCLA, University of California, Los Angeles Loneliness score; Bold: Significance.

**Table 2:** Inflammatory markers and scores at 3 and 6 months.

	Hurley II (*n =* 18)	Hurley III (*n =* 19)	Total (*n =* 37)	*p*-Value
	Measurements at 3 months			
ESR (mm/h), Median (IQR)	12 (7–18)	26 (18–36)	18 (8–26)	**0.001**
CRP (mg/L), Median (IQR)	4 (2–5)	4 (1–10)	4 (2–6)	0.41
DLQI, Median (IQR)	5 (2–10)	9 (2–16)	5 (2–15)	0.23
HADS, Median (IQR)	6.5 (5–8.5)	9 (5–10)	7 (5–9)	0.32
UCLA, Median (IQR)	32 (29–39)	33 (30–42)	33 (29–40)	0.59
	Measurements at 6 months			
ESR (mm/h), Median (IQR)	10 (5–15)	19 (11–38)	15 (7–21)	**0.005**
CRP (mg/L), Median (IQR)	2 (2–4)	5.4 (1–10)	3 (2–6)	0.14
DLQI, Median (IQR)	4 (1–9)	9 (2–12.5)	5 (2–10)	0.29
HADS, Median (IQR)	6 (5–8)	7 (4–9)	6 (4–9)	0.61
UCLA, Median (IQR)	31 (29–39)	35 (31–43)	35 (29–43)	0.29

Abbreviations: ESR, erythrocyte sedimentation rate; CRP, C-reactive protein; DLQI, Dermatology Life Quality Index; HADS, Hospital Anxiety and Depression Scale; UCLA, University of California, Los Angeles Loneliness score; Bold: Significance.

**Table 3:** Correlations among scores (DLQI, UCLA, and HADS) and inflammatory markers (ESR and CRP).

	DLQI	HADS	UCLA
	Spearman’s rho (*p*-Value)		
	Baseline		
ESR (mm/h)	0.35 (0.03)	0.20 (0.22)	0.15 (0.35)
CRP (mg/L)	0.18 (0.27)	0.02 (0.87)	−0.03 (0.85)
	At 3 months		
ESR (mm/h)	0.16 (0.32)	0.23 (0.17)	0.15 (0.35)
CRP (mg/L)	0.27 (0.1)	0.23 (0.16)	0.15 (0.36)
	At 6 months		
ESR (mm/h)	0.19 (0.25)	0.13 (0.43)	0.20 (0.22)
CRP (mg/L)	0.20 (0.21)	0.08 (0.61)	0.03 (0.82)

Abbreviations: ESR, erythrocyte sedimentation rate; CRP, C-reactive protein; DLQI, Dermatology Life Quality Index; HADS, Hospital Anxiety and Depression Scale; UCLA, University of California, Los Angeles Loneliness score.

**Table 4:** Results of univariate and multivariable mixed effects models with the scores (UCLA, DLQI, and HADS) as dependent variables and serum inflammatory markers as independent variables, adjusted for gender, age, treatment, BMI, IHS4, and smoking status.

	Univariable Analysis			Multivariable Analysis		
	Estimate β	*p*-Value	95% CI	Estimate β	*p*-Value	95% CI
Response Variable: DLQI						
ESR(mm/h)	0.15	**<0.001**	(0.08, 0.23)	0.14	**0.001**	(0.05, 0.22)
CRP(mg/L)	0.27	**0.01**	(0.05, 0.50)	0.25	**0.02**	(0.02, 0.48)
Response Variable: HADS						
ESR(mm/h)	0.03	**0.01**	(0.008, 0.070)	0.02	0.08	(−0.004, 0.05)
CRP(mg/L)	0.03	0.32	(−0.04, 0.11)	0.03	0.37	(−0.04, 0.11)
Response Variable: UCLA						
ESR(mm/h)	0.12	**0.004**	(0.04, 0.21)	0.11	**0.01**	(0.02, 0.20)
CRP(mg/L)	0.04	0.69	(−0.18, 0.27)	0.03	0.79	(−0.20, 0.26)

Abbreviations: CI, confidence interval; ESR, Erythrocyte Sedimentation Rate; CRP, C-reactive protein; DLQI, Dermatology Life Quality Index; HADS, Hospital Anxiety and Depression Scale; UCLA, University of California, Los Angeles Loneliness score; Bold: Significance.

## Data Availability

The raw data supporting the conclusions of this article will be made available by the authors on request.
